# Intraoperative Biologization of β-TCP and PCL-TCP by Autologous Proteins

**DOI:** 10.3390/jfb16090340

**Published:** 2025-09-09

**Authors:** Andrea Sowislok, Gerrit Gruber, Farnusch Kaschani, Markus Kaiser, Eleftherios Papaeleftheriou, Marcus Jäger

**Affiliations:** 1 Chair of Orthopedics and Trauma Surgery, University of Duisburg-Essen, 45147 Essen, Germany; andrea.sowislok@uni-due.de (A.S.); e.papaeleftheriou@contilia.de (E.P.); 2 Department of Orthopedics, Trauma and Reconstructive Surgery, St. Marien Hospital, 45468 Mülheim a. d. Ruhr, Germany; g.gruber@contilia.de; 3 Analytics Core Facility Essen (ACE), University of Duisburg-Essen, 45141 Essen, Germany; farnusch.kaschani@uni-due.de; 4 Chemical Biology, Faculty of Biology, University of Duisburg-Essen, 45141 Essen, Germany; markus.kaiser@uni-due.de

**Keywords:** bone regeneration, human proteome, bone substitutes, intraoperative protein adsorption, proteomics, bioactivation, surgical site released tissue, total hip arthroplasty

## Abstract

Protein adsorption on orthopedic biomaterials during the initial intraoperative contact critically influences biological responses and osseointegration. Osteoconductive grafts such as β-tricalcium phosphate (β-TCP) and poly (ε-caprolactone)-β-TCP (PCL-TCP) can be functionally activated by exposure to autologous tissue. However, the composition and relevance of the resulting protein layer still remain unclear. In this study involving 10 patients undergoing primary total hip arthroplasty, β-TCP and PCL-TCP samples were incubated both in the femoral medullary cavity and within a surgical tissue collector harvesting autologous tissue (blood, bone fragments, muscle, and fat). Surface morphology was assessed microscopically, and protein adsorption was characterized via high-resolution LC-MS/MS with subsequent bioinformatics and statistical analysis. Both materials adsorbed over 2000 different autologous proteins. β-TCP showed higher overall protein concentrations, while PCL-TCP demonstrated greater proteomic diversity and incubation method-dependent shifts in protein profiles, influenced by surface roughness and wettability. Samples incubated in the tissue collector exhibited less protein variability and smaller material-specific differences compared to incubation in the femoral cavity, particularly for PCL-TCP. Predominant proteins were linked to immune regulation, stress response, and protein metabolism. These findings emphasize the impact of material properties and incubation environment on protein adsorption, with ex vivo incubation leading to more consistent protein adsorption patterns.

## 1. Introduction

Under healthy conditions, bone tissue has a remarkable regeneration capacity without scar formation [1]. However, clinical situations such as trauma, post tumor resection, or in aseptic implant failure, may lead to large bone defects where natural healing is impaired [2,3]. Defects that fail to heal spontaneously, or even after surgical stabilization, are referred to as critical size bone defects and are characterized by delayed union, non-union/pseudarthrosis. In these complicated cases, further surgical intervention might be required. This includes the application of bone substitutes to enhance and accelerate bone regeneration [4]. An ideal bone graft should mimic native bone, is biocompatible, bioresorbable, osteoinductive and/or osteoconductive, while also easy to handle and cost-effective [5]. Osteoinductivity refers to the stimulation of MSC migration, proliferation, and differentiation into preosteoblasts, whereas osteoconductivity supports bone growth along surfaces structure (e.g., pores or channels) [6]. Here, autologous bone transplantation still remains the gold standard, though limited by donor site morbidity, pain, limited availability, and the need for additional surgery [7,8]. Allografts and xenografts lack osteogenic capacity and carry risks such as immune rejection, disease transmission, and offer lower osteoactivity [9]. Synthetic bone grafts, especially ceramics like β-tricalcium phosphate (β-TCP), are widely used in orthopedics and dentistry [10,11,12]. Mimicking the hydroxyapatite phase of bone, β-TCP is osteoconductive [13]. Though insoluble physiologically, it is fully resorbed and replaced by bone within 0.5–1.5 years or longer [14]. However, brittleness limits its use in load-bearing applications [15]. In contrast, poly (*ε*-caprolactone) (PCL) is a thermoplastic polymer with higher mechanical stability, able to retain strength under deformation [16]. Its low melting point (~60 °C) allows for customized scaffold fabrication via 3D printing, electrospinning, or injection molding [17]. PCL degrades over two years through non-enzymatic hydrolysis followed by cell-mediated breakdown into non-toxic, low-molecular-weight fragments [18]. However, PCL’s hydrophobic nature hinders cell adhesion [19]. To address this, surface modifications (e.g., protein adsorption, mineralization), the addition of growth factors or progenitor cells have been realized [16,17,20]. It has been shown, that combining PCL with ~20% β-TCP improves its mechanical and degradative properties [21,22,23,24].

Recent studies show that osteoconductive scaffolds like β-TCP and PCL-TCP can be biologically activated intraoperatively through exposure to surgical site-released tissue (SSRT) using surgical tissue collectors [25,26,27,28]. Intraoperative biologization is a promising strategy to enhance implant integration. However, detailed proteomic analyses of protein adsorption onto bone substitutes under clinical conditions are lacking. In particular, the impact of intraoperative handling and material properties on the composition of the adsorbed protein layer remains poorly understood, despite its critical role in early cellular responses and osseointegration. In this study, we present the first comprehensive proteomic characterization of autologous protein adsorption onto β-TCP and PCL-TCP immediately following intraoperative patient contact. We compare two clinical incubation methods -direct femoral medullary cavity exposure and indirect incubation using a tissue collector-and assess their impact on the amount and diversity of adsorbed proteins, addressing a key gap in the current literature.

## 2. Materials and Methods

### 2.1. Patients

Our study included 10 adult patients (8 females, 2 males, mean age of 68.8 ± 10.6 years) with advanced hip osteoarthritis scheduled for primary total hip replacement. Exclusion criteria included septic conditions, active neoplasms or other consuming diseases (such as autoimmune disorders) and coagulopathy. The study was approved by the Ethics Committee of the Medical Faculty at the University of Duisburg-Essen, Germany (No. 22-10667-BO), and conducted in accordance with the Declaration of Helsinki. All probands provided informed consent.

### 2.2. Sample Collection

An anterolateral approach to the hip joint was performed following the Hardinge-Bauer technique [29,30]. After femoral neck resection, samples of the commercial bone substitute β-TCP (3000–5000 µm, 10 cc; Cerasorb^®^ M, Curasan AG, Kleinostheim, Germany) and PCL-TCP (80% PCL & 20% TCP composite material (50 mm × 50 mm × 1.25 mm); Osteomesh™, Osteopore Pty Ltd., Singapore) were implanted into the opened femoral medullary cavity (FC group) and removed after 2 min in situ. Additional samples of these biomaterials were incubated in the inner filter of a surgical suction handle device (BoneFlo^®^, TissueFlow GmbH, Essen, Germany) (BF group) for 20 min with the patient’s surgical site released tissue (blood, bone/bone marrow fragments, muscle and fat) collected during surgery. The harvested biomaterials from the FC group and the entire suction handle containing the BF group were separately transported to the laboratory under sterile conditions at 4 °C for further processing.

### 2.3. Removal of the Adsorbed Protein Layer

In the BF group β-TCP granules and PCL-TCP membrane were removed from the tissue collector under sterile conditions. PCL-TCP membranes were cut into smaller pieces (BF and FC group). All bone substitutes were washed with physiological saline solution. A small sample (approx. 500 mg) was taken, weighed, and transferred to a 5 mL Eppendorf tube for protein elution. The bone substitutes were then washed sequentially with 1 mL of the following buffers for 5 min, with occasional vortexing: (i) buffer A (4% SDS, 1 M DTT, 0.1 M Tris-HCl pH 7.6), (ii) buffer B (4% SDS, 1 M DTT, 1 M NaCl, 0.1 M Tris-HCl pH 7.6) and (iii) buffer C (4% SDS, 0.1 M DTT, 1 M NaCl, 0.1 M Tris-HCl pH 7.6). The solutions were pooled and mixed with 600 µL 1 M iodoacetamide in 2 M Tris-HCl pH 8.6.

### 2.4. Protein Quantification and Proteome Analysis

Before protein quantification using a modified BCA assay (Thermo Fisher Scientific, Rockford, IL, USA), we performed TCA precipitation to remove interfering substances, as previously described [31,32].

Sample preparation and proteome analysis were conducted as previously described [33]. Briefly, after SP3 trypsin digestion, proteome analysis was conducted using LC-MS/MS on an Orbitrap Elite instrument (Thermo, Waltham, MA, USA) coupled with an EASY-nLC 1000 liquid chromatography system (Thermo). The Orbitrap analyzer (FTMS; Fourier transform mass spectrometry) scanned precursor ions in the *m*/*z* range of 300–1800 at a resolution of 60,000, with the internal lock mass option enabled using polysiloxane (lock mass: 445.120025 *m*/*z*). The RAW spectra were processed using the Andromeda search engine within MaxQuant (version 2.0.3.0.) with default settings, label-free quantification, and match-between-runs enabled. MS/MS data were searched against the Uniprot Homo sapiens reference database (UP000005640_9606_OGPP.fasta, 20,589 entries, downloaded on 10 January 2022), alongside a contaminant database. Further analysis was conducted using Perseus (version 1.6.10.0).

### 2.5. Proteomic Data Annotation and Bioinformatics Analysis

The proteome was compared with the plasma and the red blood cell proteomes based on previously published data using the merge function in R-studio (version 4.2.0). A reference list for the plasma proteome included 945 proteins (≥10 peptide spectrum matches and ≥2 peptides) by Farrah et al. [34], 1175 proteins by Anderson et al. [35] and 713 proteins (≥2 peptides) by Geyer et al. [36]. The red blood cell proteome reference list included 2309 proteins (≥2 peptides) by Bryk et al. [37]. Pathway enrichment analysis was performed using the Reactome online tool [38,39]. Pathways with a false discovery rate (FDR) below 0.05 were considered significantly enriched.

### 2.6. Statistics and Data Visualization

Statistical analysis was conducted using RStudio (version 4.2.0, 22 April 2022, R Foundation for Statistical Computing). Data normality was assessed using the Shapiro–Wilk test. Non-normally distributed continuous data are presented as mean ± standard deviation (SD) and were compared using the Wilcoxon signed rank test for dependent samples. Surface roughness was analyzed using the paired Wilcoxon test. Molecular weight distribution was analyzed using the Mann–Whitney U test, with *p*-values < 0.05 considered statistically significant. To identify differentially abundant proteins, the following pairwise comparisons were made: (1) TCP and PCL incubated in tissue collector (BF), (2) TCP and PCL incubated in femoral medullary cavity (BC), (3) PCL incubated in BF and in BC, (4), TCP incubated in BF and in BC. Differentially abundant proteins in each group were defined based on the following criteria: Proteins identified in at least 60% of samples in at least one group and nominally significant difference in protein abundance (unadjusted *p* < 0.05).

Since the proteomic data were dependent within comparisons and were not normally distributed in most cases, the paired Wilcoxon test with continuity correction was used due to the small sample size (n < 40) and potential ties. Proteins occurring in less than 20% of both comparison groups were excluded from the statistical analysis as invalid.

Pie diagrams and bar charts were created in Microsoft Excel (MS Office Professional Plus 2016), while volcano- and boxplots were generated using Prism v.9.5.1.

### 2.7. Surface Roughness Analysis

Surface roughness was analyzed optically using a VHX 7000 digital microscope (Keyence, Neu-Isenburg, Germany) equipped with a 50–500× zoom lens (VH-Z50T) and a ring light (OP-88135). The roughness was evaluated usin g the surface roughness parameter Sa, which represents the arithmetic mean height deviation from the mean plane [40].

To determine the surface roughness, TCP and PCL specimens were analyzed before and after protein elution. For each sample, a depth of field image was captured at three different positions at 500× magnification. The roughness was measured on an area of 0.2 mm^2^ using a Gaussian filter without applying additional S and L filters and the mean value was calculated.

### 2.8. Scanning Electron Microscopy (SEM)

For scanning electron microscopy, bone substitutes from the BF and FC groups were fixed for 3 h at RT in 2.5% glutaraldehyde and 4% formaldehyde in PHEM buffer (pH 6.9; 60 mm PIPES, 25 mm HEPES, 10 mm EGTA, and 4 mm MgSO4), following previously described washing procedure. After fixation, samples were washed (2 × PHEM, 3 × dest. H2O) and dehydrated through a graded ethanol series (30%, 50%, 70%, 80%, 97%, and 3 × 100%) using a BioWave Pro+ microwave system (Ted Pella, Redding, CA, USA) at 250 W for 40 s at 20 °C. The dehydrated samples were dried using a critical point dryer (EM CPD 300 Leica, Wetzlar, Germany) mounted on a carbon-coated stub and sputter-coated with a 15 nm thick platinum/palladium layer (EM ACE 600, Leica, Wetzlar, Germany). SEM images were acquired with a FIB-SEM (Crossbeam 540, Carl Zeiss, Jena, Germany) operated at an accelerating voltage of 1.5 kV and a beam current of 2 nA in analytical column mode.

## 3. Results

### 3.1. Surface Characteristics of Bone Substitutes After Intraoperative Exposure

**Figure 1:**
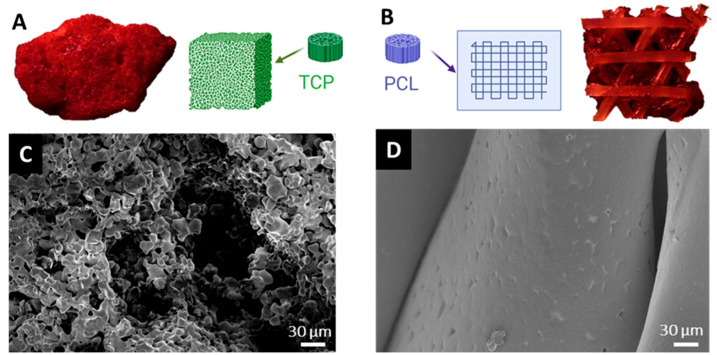
Schematic and microscopic overview of bone substitutes. (**A**): Ceramic β-TCP granule and (**B**): 3D-printed PCL-TCP membrane fragment after intraoperative incubation, showing visible blood coloration. (**C**): SEM image of β-TCP reveals a highly porous surface. (**D**): SEM image of PCL-TCP shows a smooth, non-porous surface.

**Figure 2:**
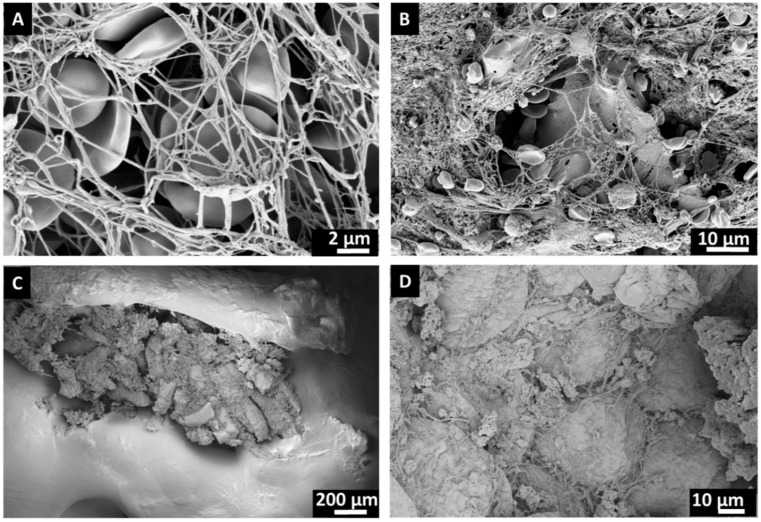
SEM images of bone substitutes after intraoperative incubation. (**A**): Red blood clots, (**B**): bone fragments, (**C**): muscle tissue and (**D**): adipose tissue adhering to the biomaterial surface.

After the initial in situ introduction of the bone substitute in either BF or FC and subsequent washing with physiological saline solution, both β-TCP and PCL-TCP bone substitutes exhibited visible blood staining, with no macroscopic differences observed between the materials or the incubation methods (Figure 1). SEM analysis revealed surface coverage with red blood clots as well as remnants of bone fragments, muscle and adipose tissue (Figure 2).

### 3.2. Impact of Protein Elution and Incubation Method on Bone Substitute Surface Roughness

**Figure 3:**
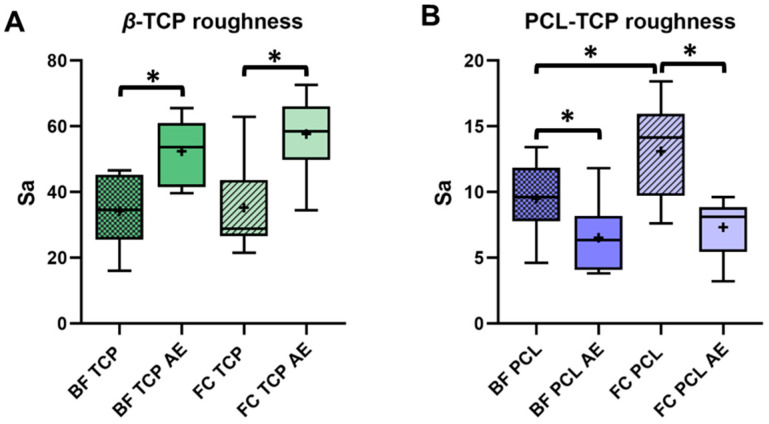
Surface roughness (Sa) of bone substitutes before and after protein elution. Boxplots illustrate the Sa parameter measured before and after elution (AE) of adsorbed proteins. (**A**): β-TCP surface roughness following incubation in the tissue collector (BF) and femoral medullary cavity (FC), shown both pre- and post-elution. (**B**): PCL-TCP surface roughness under the same conditions. Statistical significance is indicated by * (*p* < 0.05).

Roughness measurements revealed a higher surface roughness for β-TCP compared to PCL-TCP, which was consistent with the SEM micrographs showing a smooth PCL-TCP and a porous β-TCP structure (Figure 1). For β-TCP, a significant increase in surface roughness was observed after protein elution, with Sa values increasing from 34.2 ± 11.5 to 52.4 ± 9.8 (BF, *p* = 0.005) and from 35.3 ± 13.7 to 57.7 ± 11.7 (FC, *p* = 0.006). In contrast, the roughness of PCL-TCP decreased significantly with Sa values dropping from 9.5 ± 2.7 to 6.5 ± 2.6 (BF, *p* = 0.017) and from 13.1 ± 3.6 to 7.3 ± 2.2 (FC, *p* = 0.004) (Figure 3). While no statistical difference in the roughness of β-TCP was observed between incubation methods (*p* = 0.625), the surface roughness of PCL-TCP was significantly higher for samples incubated in femoral the femoral medullary cavity (FC) compared to those in the tissue collector (BF) FC (*p* = 0.037).

### 3.3. Quantification of the Adsorbed Protein Layer on β-TCP and PCL-TCP

**Figure 4:**
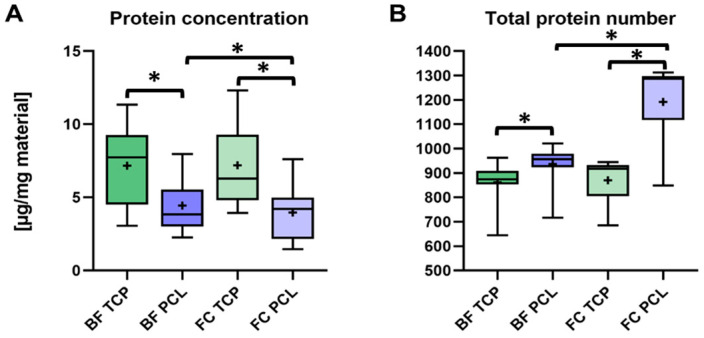
Protein adsorption on bone substitutes: concentration and diversity. (**A**): Protein concentration (µg/mg material) of eluted proteins from β-TCP and PCL-TCP following incubation in the tissue collector (BF) and the femoral medullary cavity (FC). (**B**): Total number of unique proteins identified on each material under both incubation conditions. Statistical significance is indicated by * (*p* < 0.05).

Both bone substitutes showed similar protein concentrations when incubated in BF and FC: 7.17 ± 2.67 µg/mg and 7.19 ± 2.73 µg/mg for β-TCP and 4.44 ± 1.8 µg/mg and 3.96 ± 1.91 µg/mg for PCL-TCP, respectively (Figure 4A). The influence of the incubation method was not statistically significant for β-TCP (*p* = 0.967), but it was statistically significant for PCL-TCP (*p* = 0.021). In general, the protein concentration adsorbed to β-TCP was significantly higher compared to PCL-TCP in both groups (BF: *p* = 0.019; FC: *p* = 0.006).

### 3.4. Proteomic Profile and Protein Origin in β-TCP and PCL-TCP Samples

**Figure 5:**
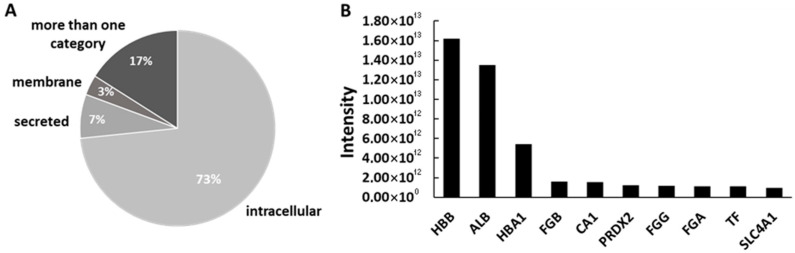
Subcellular origin and abundance of adsorbed proteins. (**A**): Subcellular localization of proteins adsorbed to bone substitutes, showing a predominance of intracellular proteins. (**B**): Top 10 most abundant proteins across all samples, with hemoglobin and albumin being the most highly enriched. An extensive list of all proteins, along with the relevant annotations—including subcellular localization, signal intensity, and plasma proteome association—is provided in the Supporting Information (see Supplementary Materials Table S1).

Proteome analysis using LC-MS/MS identified over 2000 different protein groups, covering a large dynamic range of log_2_(x) from 18 to 40. After refinement (≥2 peptides, ≥60% of all samples within a group) the final count was 1410 unique proteins. Depending on the reference set used, 16% to 30% of these proteins were annotated as plasma proteins, while 55% were classified as part of the red blood cell proteome. The majority of the identified proteins were of intracellular origin (73%, n = 1032), followed by secreted proteins (7%, n = 102), membrane-associated proteins (3%, n = 47), and 17% (n = 227) that could belong to multiple categories depending on the isoform (Figure 5A). Among the 10 most abundant proteins, we identified hemoglobin, serum albumin, fibrinogen and other plasma proteins, as well as proteins from red blood cells (Figure 5B).

When examining the average protein count per eluate across the four groups, a significantly higher protein count was observed for PCL-TCP with 936 ± 84 proteins in BF (*p* = 0.007) and 1192 ± 152 (*p* = 0.001) proteins in FC, compared to β-TCP, which had 937 ± 84 proteins in BF and 870 ± 88 proteins in FC. Additionally, the protein count was significantly higher for PCL-TCP incubated in FC than in BF (*p* = 0.002), while no significant difference was found for β-TCP (*p* = 0.447) (Figure 4B).

### 3.5. Material-Specific Differences in Protein Adsorption Profiles

#### 3.5.1. Selective Protein Adsorption on β-TCP and PCL-TCP Following Surgical Tissue Collector (BF) Incubation

**Figure 6:**
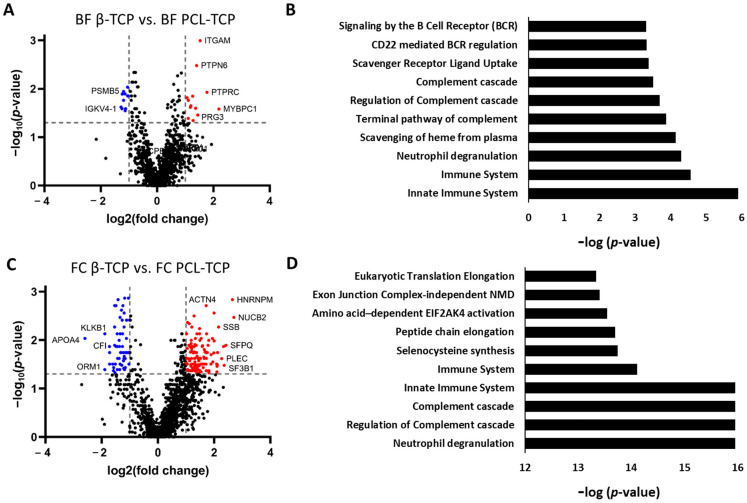
Material-specific protein adsorption profiles and enriched pathways following BF and FC incubation. (**A**): Volcano plot displaying significantly differentially adsorbed proteins between β-TCP and PCL-TCP after incubation in the surgical tissue collector (BF). Proteins with a log_2_ fold change > ±1 and *p*-value < 0.05 are highlighted, revealing distinct material-dependent adsorption profiles. (**B**): Top 10 significantly enriched pathways based on differentially adsorbed proteins, ranked by −log(*p*-value). (**C**): Volcano plot of differentially adsorbed proteins between β-TCP and PCL-TCP after incubation in the femoral medullary cavity (FC), with the same significance thresholds applied. (**D**): Corresponding top 10 enriched pathways, ranked by −log(*p*-value). NMD = Nonsense Mediated Decay. An extensive list of all proteins, including their corresponding *p*-values and Reactome analysis, is provided in the Supporting Information (see Supplementary Materials Table S2).

Out of the 1089 proteins analyzed for surface adsorption, only 71 displayed a statistically significant difference in abundance between β-TCP and PCL-TCP. Applying a fold change (fc) threshold of |log_2_fc| > 1 narrowed this list to 25 proteins. Among these, 13 proteins were more abundant on the PCL-TCP surface. Notably, proteins enriched on PCL-TCP also exhibited higher fc values; for example, PTPRC and MYBPC1 showed log_2_fc values of 1.8 and 2.2, respectively, while ATRN, more abundant on β-TCP, showed a log_2_fc of −1.3 (Figure 6A).

An evaluation of the molecular weight (MW) distribution among these 25 proteins revealed a trend towards higher MW in proteins adsorbed on PCL-TCP (mean: 126.9 kDa) compared to those on β-TCP (mean: 55.8 kDa), though this difference did not reach statistical significance (*p* = 0.0523).

Functional enrichment analysis revealed that most significantly different proteins are involved in immune-related pathways, including neutrophil degranulation, the complement cascade, and B-cell receptor signaling, all key components of immune system processes (Figure 6B).

#### 3.5.2. Pronounced Material-Dependent Adsorption Differences After Femoral Medullary Cavity (FC) Incubation

Following FC incubation, substantial differences in protein adsorption between β-TCP and PCL-TCP were observed. Of the 1517 proteins analyzed, 238 showed statistically significant differences in abundance, with 193 meeting the fold change (fc) threshold (|log_2_fc| > 1). Among these, 62 proteins (32%) were more abundant on β-TCP, while 131 proteins (68%) were enriched on PCL-TCP.

The proteins significantly enriched on PCL-TCP generally exhibited higher fc values. Sixteen proteins displayed a log_2_fc > 2 on PCL-TCP, whereas only one protein showed a log_2_fc < −2 on β-TCP. The molecular weight distribution of these differentially abundant proteins was comparable between the two materials (67.3 kDa for β-TCP vs. 78.6 kDa for PCL-TCP), with no statistically significant difference (*p* = 0.5622) (Figure 6C).

Pathway enrichment analysis revealed patterns similar to those observed under BF incubation, predominantly involved in immune-related processes such as neutrophil degranulation, the complement cascade, and B-cell receptor signaling. However, in contrast to BF incubation, these pathways exhibited lower *p*-values and false discovery rates (FDR), indicating a more robust association of immune system pathways with the protein adsorption profile post-MR incubation (Figure 6D).

### 3.6. Incubation-Dependent Protein Adsorption Profiles on Bone Substitutes

#### 3.6.1. Protein Adsorption on β-TCP Is Minimally Affected by Incubation Method

**Figure 7:**
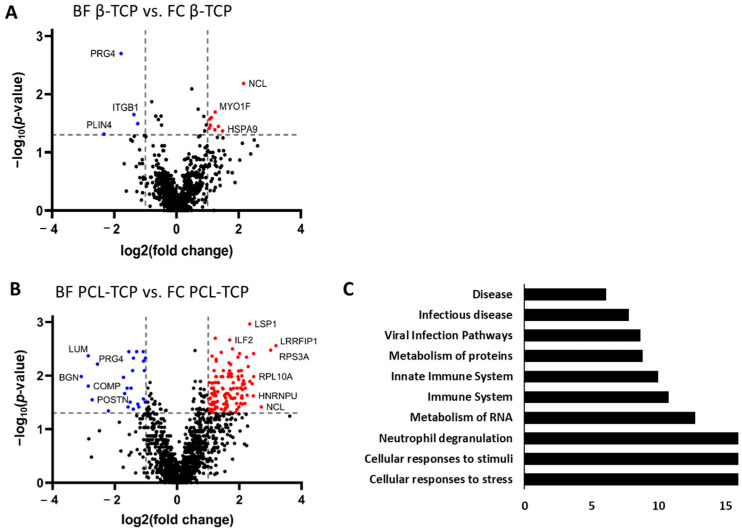
Incubation-dependent protein adsorption profile on *β*-TCP and PCL-TCP. (**A**): Volcano plot showing proteins significantly differentially adsorbed on β-TCP after incubation in the surgical tissue collector (BF) versus the femoral medullary cavity (FC). Proteins with a log_2_ fold change > ±1 and *p*-value < 0.05 are highlighted, indicating incubation-specific alterations in the β-TCP protein composition. (**B**): Volcano plot of proteins differentially adsorbed on PCL-TCP under the same conditions. (**C**): Top 10 significantly enriched pathways associated with the differentially adsorbed proteins, ranked by –log(*p*-value). An extensive list of all proteins, including their corresponding *p*-values and Reactome analysis, is provided in the Supporting Information (see Supplementary Materials Table S3).

Protein adsorption profiles on β-TCP showed minimal differences between BF and FC incubation. Out of 1029 proteins analyzed, only 23 displayed statistically significant changes in abundance, and applying the fc threshold (|log_2_fc| > 1) reduced this number to 13 proteins. Among these, 9 proteins were more abundant following FC incubation. Conversely, several notable proteins were enriched after BF incubation, including the proteoglycans PRG4 (log_2_fc = −1.78) and ASPN (log_2_fc = −1.24), as well as the adipocyte-associated protein PLIN4 (log_2_fc = −2.33). The average molecular weight of significantly different proteins tended to be higher in the BF condition (104.7 kDa) compared to FC (71.4 kDa), though this difference did not reach statistical significance (*p* = 0.1986) (Figure 7A). Pathway enrichment analysis did not yield significant results due to exceeding the FDR threshold (FDR > 0.07), suggesting that the observed differences are either biologically subtle or involve proteins not strongly clustered into known functional pathways.

#### 3.6.2. Incubation Method Strongly Alters Protein Adsorption on PCL-TCP

In contrast to the relatively stable protein adsorption profile observed on β-TCP, the method of incubation significantly influenced the protein adsorption profile on PCL-TCP. Of the 1365 proteins analyzed, 192 exhibited statistically significant differences in abundance between BF and FC conditions, with 146 proteins meeting the fc threshold (|log_2_fc| > 1). FC incubation had a notably stronger effect, resulting in 121 proteins being significantly enriched, compared to only 25 proteins enriched following BF incubation (Figure 7B). Proteins enriched under BF conditions were predominantly involved in extracellular matrix (ECM) organization, including key structural and regulatory proteins such as CD47, FBLN1, and EFEMP1. Among these, several ECM proteins specific to bone and cartilage displayed the largest negative fold changes -indicating preferential adsorption during FC incubation- including biglycan (BGN, log_2_fc = −3.08), cartilage oligomeric matrix protein (COMP, −2.86), lumican (LUM, −2.56), and decorin (DCN, −2.21). Among the 30 proteins with log_2_fc > 2 enriched under FC conditions, 11 were associated with cellular stress responses, including six ribosomal subunit proteins (RPL10A, RPL6, RPS13, RPS18, RPS19, RPS3A), suggesting an activation of translational or ribosomal pathways. A comparison of molecular weight distributions revealed a statistically significant difference: proteins adsorbed following BF incubation exhibited a higher average molecular weight (69.7 kDa) than those adsorbed in FC (49.2 kDa; *p* = 0.0365). Pathway enrichment analysis of all 146 significantly different proteins identified numerous enriched pathways. When grouped by overarching biological processes, the most enriched categories included cellular responses to stimuli and stress, neutrophil degranulation, RNA and protein metabolism, and general immune system functions (Figure 7C).

## 4. Discussion

### 4.1. Protein Adsorption as a Key Regulator of Implant Integration

Once an implant is placed into the body, its surface is immediately exposed to the biological microenvironment on the insertion site of the patient, which includes cells, proteins, and other components of body fluids. Intraoperative injury to blood vessels and subsequent bleeding result not only in implant wetting and blood clot formation, but also in the rapid adsorption of a diverse array of proteins onto the implant surface [41]. This initial protein layer is critical, as it acts as a biological interface for cellular attachment, initiates the inflammatory cascade and plays a key role in guiding subsequent in situ processes such as cell adhesion, migration, proliferation, differentiation, biomineralization, and thus ultimately bone remodeling and osseointegration. Despite its importance, the exact composition of this protein layer immediately after the first patient contact still remains largely unknown [42]. Protein adsorption on biomaterial surfaces is influenced by numerous factors, including both the biochemical properties of the proteins as well as the physicochemical characteristics of the implant surface [43,44,45].

### 4.2. Material-Dependent Protein Adsorption: β-TCP vs. PCL-TCP

One aim of our study was to investigate the material-specific differences in the adsorption of intraoperative proteins to the bone substitutes β-TCP and PCL-TCP, which differ in their wettability and porosity. We found that although the overall protein concentration per mg of sample was higher for β-TCP, a greater diversity of proteins was adsorbed to PCL-TCP—an observation that may appear contradictory at first glance. However, when considered individually, both findings are logical: the higher protein concentration on β-TCP can be attributed to its greater porosity and surface roughness, which increase the available surface area and enhance protein loading [46]. Conversely, hydrophobic materials like PCL-TCP tend to adsorb larger protein quantities due to favorable hydrophobic interactions, albeit with lower overall selectivity [43]. Importantly, high total adsorption does not imply high protein diversity. After protein removal, the surface roughness of β-TCP increased, while that of PCL-TCP decreased. This likely reflects the re-exposure of clogged pores in the porous β-TCP structure, leading to greater surface irregularity, while on the smoother PCL-TCP surface, the removal of protein aggregates results in a more uniform topography. While the role of wettability in protein adsorption is well established, the influence of the surface topography is often underestimated [47]. The literature on this topic is also inconsistent: some studies report no change [48,49] or even a decrease [50] in protein adsorption with increasing roughness, whereas others observe enhanced adsorption [46,51,52]. This variability can be explained by the interplay between protein structure and surface features. For instance, roughness favors the adsorption of elongated proteins like fibrinogen (340 kDa) due to higher packing density resulting from end-on orientation and possible multilayer formation, whereas compact proteins like albumin (65 kDa) are less affected due to side-on binding [52,53,54]. Pores may further promote selective adsorption of smaller proteins via size-exclusion. Nanopore confinement promotes nucleation and multilayer adsorption, increasing protein loading beyond monolayer levels [51,55]. Supporting this, our molecular weight analysis showed a non-significant trend toward smaller proteins on β-TCP after BF incubation, possibly reflecting this size-selective adsorption. Wettability also governs protein–surface interactions: hydrophobic surfaces promote adsorption through entropy gains from unfolding and structured water displacement, while hydrophilic or charged surfaces favor electrostatic and van der Waals interactions, influenced by the zeta potential of both surface and protein [43,44]. β-TCP, with its calcium and phosphate surface groups, likely promotes strong electrostatic interactions, contributing to its high protein binding capacity [56,57]. Surface roughness itself can increase hydrophilicity [54], and protein adsorption can further modify surface wettability depending on which protein domains are exposed [58]. From a thermodynamic perspective, protein adsorption is driven by a combination of enthalpic and entropic factors. Referring to hydrophobic materials like PCL-TCP, entropy dominates due to water release and protein unfolding, while on ionic surfaces like β-TCP, enthalpic interactions—such as electrostatics—play a larger role [59,60,61]. Notably, adsorption is thought to be most favorable when the ratio of polar to dispersive surface energy components approaches unity, offering a balance between enthalpic and entropic contributions [62].

### 4.3. Impact of Incubation Conditions on Protein Adsorption

The second aim of our study was to assess how incubation conditions affect human protein adsorption. We compared two methods: a short, high-temperature condition (FC: 2 min at 37 °C, in situ) and a longer, room-temperature condition (BF: 20 min at 25 °C, ex vivo). These parameters influence the mobility, folding and dynamics of the surface interaction of proteins [63]. Material-specific differences were more pronounced under FC incubation, with PCL-TCP exhibiting higher protein quantity and diversity. In contrast, BF conditions resulted in more similar protein profiles between the materials, with less significant differences. β-TCP showed stable adsorption across both conditions, while PCL was more sensitive—suggesting that hydrophobic surfaces are more affected by environmental factors such as temperature and time. Increased temperatures increase the diffusivity of proteins and promote the release of structured water, resulting in entropy gains that facilitate adsorption [63,64,65]. Hydrophobic surfaces like PCL benefit from this effect, as higher temperatures promote protein unfolding, enhancing surface contact. This aligns with previous findings that thermally induced unfolding enhances adsorption on weakly interactive surfaces [66]. Incubation time also had a major impact, as reflected in the Vroman’s effect, which describes the time-dependent replacement of initially adsorbed, low affinity proteins by larger high affinity ones. In complex mixtures, proteins such as HSA bind first, but are later displaced by fibrinogen, fibronectin or complement factors [67,68,69]. This dynamic exchange is influenced by surface properties and exposure duration ultimately leading to a thermodynamically optimized protein layer [70]. Under BF conditions (20 min, RT), the extended incubation allowed diffusion-driven protein exchange, supporting the idea that the final composition of the adsorbed protein layer reflects thermodynamic equilibrium rather than initial kinetic trapping. Molecular weight analysis revealed significantly more high-molecular-weight proteins on both PCL and TCP after BF incubation compared to FC, which is consistent with Vroman dynamics.

### 4.4. Comparative Insights from Intraoperative Proteomes of Bone Substitutes and Hip Stems

In our previous studies, we investigated protein adsorption on titanium hip stems (BiCONTACT™, B.Braun Aesculap) after direct 2- or 5 min in situ contact with the femoral canal of patients undergoing total hip arthroplasty [31,32]. Similarly, when examining protein adsorption on the bone substitutes β-TCP and PCL-TCP in the current study, we observed parallels with our previous findings. The total number of adsorbed proteins was comparable in both cases, exceeding 2000, with over 70% originating from intracellular proteins. Here, hemoglobin and albumin were again the proteins with the highest abundance. However, the bone graft proteome contained a significantly higher proportion of proteins from the plasma (16–30% vs. 4–18%) and red blood cell (55% vs. 25%) compared to the hip stem proteome [32]. This is likely due to a higher presence of coagulated blood on the bone substitutes, as evidenced by SEM micrographs showing macroscopic blood discoloration and red blood cells on the surface. Additionally, our current study highlighted the strong involvement of immune system-related biological signaling pathways, such as neutrophil degranulation and the complement cascade, which aligns with our previous findings [32].

### 4.5. Study Limitations and Future Clinical Implications

One limitation of our study is the relatively small cohort of 10 patients, which may affect the generalizability of the proteomic findings due to potential biological variability. To assess and minimize this risk, we calculated the coefficient of variation (CV) for each protein across patients. The CV, defined as the standard deviation divided by the mean of the measured values, reflects the relative variability in protein abundance [71]. For statistically significant proteins, CVs were consistently below 10%, demonstrating high reproducibility. Among non-significant proteins, only 1–5% showed CVs above 10%, and none exceeded 20%, suggesting low interindividual variability and a limited risk of bias. Nevertheless, we plan to validate these findings in a larger patient cohort in future studies.

A second limitation concerns the differing incubation protocols for the in situ and ex vivo conditions. In situ incubation in the femoral medullary canal was limited to two minutes at 37 °C due to ethical and clinical constraints, as prolonged exposure could increase infection risk and compromise patient safety. To investigate time-dependent effects, we included an ex vivo condition using a surgical tissue collector, with a 20 min incubation at room temperature, reflecting both the typical duration of femoral preparation during total hip arthroplasty and ambient operating room conditions. While this ex vivo model does not fully replicate the physiological environment of the femoral medullary canal, it offers a practical and ethically acceptable alternative for studying temporal protein adsorption. Moreover, it may represent a feasible strategy to biologically activate bone substitutes with autologous proteins prior to implantation, potentially reducing the need for autologous bone grafting in future clinical settings.

Although this study presents the first comprehensive intraoperative proteomic analysis of protein adsorption onto β-TCP and PCL-TCP, it remains an early-stage investigation focused on material characterization. The observed protein profiles likely influence key biological processes such as cell adhesion, proliferation, and differentiation, but the absence of functional assays limits conclusions regarding their clinical relevance. Future studies will explore how the adsorbed protein layer affects the behavior of patient-derived mesenchymal stem cells, particularly with regard to osteogenic differentiation and the expression of relevant markers. These investigations will be essential for translating our proteomic findings into meaningful clinical applications.

## 5. Conclusions

This study presents the first comprehensive proteomic analysis of intraoperative human protein adsorption onto β-TCP and PCL-TCP bone substitutes, demonstrating that biologization with autologous proteins is feasible and strongly influenced by material properties and incubation conditions. While β-TCP showed stable adsorption across conditions, PCL-TCP was more sensitive to incubation time and temperature. The use of a surgical tissue collector minimized material-specific differences, enabling a more consistent and equilibrated protein layer. These results emphasize the crucial role of surface chemistry and intraoperative handling in shaping the early biointerface and controlling the regeneration outcome. Future research should investigate how these protein layers influence the behavior of osteogenic cells in order to develop personalized, bioactive bone grafts.

## Data Availability

The mass spectrometry proteomics data for the on-bead digestions have been deposited to the ProteomeXchange Consortium via the PRIDE partner repository (https://www.ebi.ac.uk/pride/archive/ (accessed on 29 July 2025) with the dataset identifier PXD066010. During the review process the data can be accessed via a reviewer account (Username: reviewer_pxd066010@ebi.ac.uk; Password: 44703ZpT5cUI.

## Supplementary Materials

The following supporting information can be downloaded at: https://www.mdpi.com/article/10.3390/jfb16090340/s1, Table S1: annotation plasma proteome, Table S2: material specific differences, Table S3: incubation specific differences.
